# Reflections in a Surgical Ward
*The writer is a young surgeon who was operated upon for appendicitis during the quiescent period. The operation was a success, and the after-course was uneventful except for a slight complication in the shape of a mild attack of broncho-pneumonia.


**Published:** 1913-06-14

**Authors:** 


### HOSPITALS FROM THE PATIENTS' POINT OF VIEW.

(Contributions to this Section are Invited.

### Reflections in a Surgical Ward.*

Knowing, as I did, what I had to expect in the way of preparation and after-treatment, I did not anticipate, when I entered the hospital, that I should have anything material to chronicle in the way of experience that might be productive of helpful suggestion. The first day, however, showed me that the best school is that which requires personal attendance, and that the best knowledge comes not from observation on one's own cases, but from actual personal experience. No one, I imagine, looks forward with exhilaration to the preliminaries which the modern ritual of aseptic surgery prescribes in the case of every patient who has to undergo operation. I had often carried out these preliminaries on patients, and had more often supervised them; in fact, I thought that I had nothing further to learn with regard to them. Yet I found that what I had formerly regarded as an entirely trivial thing was a source of inconvenience and discomfort.

### Preparing the Patient for Operation.

The hospital had male attendants who were proficient and skilful, and who did their work with gentleness and care, but my experience taught me the value of two small points which are not often insisted upon in the schools. One is that a safety razor is an essential in shaving; it is much more easily managed by a nurse or attendant who is not a proficient barber, and it leaves the skin, unused to such preliminary denudation of hair, much less chafed and worried than does the average hospital razor, which is too often blunt. The second point is that the old-fashioned perchloride compress is an abomination. An efficient sterilisation can be secured by sponging with iodine after the skin has been carefully cleansed with soap and water, a grease-remover such as ether or benzine, and then very thoroughly dried. The strength of the iodine solution used is of some importance; so, too, is the manner in which it is applied, for if it touches very delicate skin it occasions considerable smarting, which, however, soon passes off into a feeling of comfortable warmth and glow.

It is perhaps superfluous to suggest that every hospital should have a male attendant to do these important preliminaries for male patients. In the hospitals with medical schools it is the duty of the dresser to attend to the male patients; in others the nurse is supposed to do the shaving and cleaning. The latter system is, to my mind, a mistake, and should be given up. My impressions of the night before operation do not yield anything in the way of fruitful suggestion beyond this; I lay in a quiet part, and was absolutely undisturbed by noise of any kind, so that I had a comfortable and restful sleep. The last round of the house surgeon found me quietly sleeping, and I was not awakened for temperature-charting.

### Suggested Cause of Bronchial Complications.

In the morning, the operation having been fixed for eight o'clock, I had the usual light breakfast ordered in uncomplicated appendicectomy cases, and half an hour afterwards, at my own request, walked into the theatre in dressing-gown and slippers. I found the temperature in the corridors appreciably lower than in the room I had just quitted, and that of the anæsthetist's room much too warm for my comfort. Personally, I believe that these changes of temperature have much to do in causing the subsequent bronchial complications which are unjustifiably put down to the anæsthetic. In my case the anæsthetic was given with great skill, and I experienced absolutely no discomfort of any kind which I can honestly ascribe to the drug, either immediately or remotely afterwards. When I "came to" about half an hour after the operation, I felt quite fit, somewhat hungry, but with no pain or discomfort to remind me that I had just had my appendix removed. This want of after-pain—which is, however, by no means great in appendicectomy cases, except in highly nervous individuals—I ascribe largely to the care taken with the dressing, the efficient drying of the skin adjacent to the site of operation before the adhesive plaster was put on, and the tightness with which the plaster itself was put on.

During the ensuing days, before my bronchial trouble caused any discomfort, I had nothing whatever to complain of. My nurses were the most carefully trained persons I have ever worked with, and my case was not one that demanded special nursing or that presented any features of particular difficulty. I saw friends several times each day, read books and papers, and had food at the earliest opportunity.

### The Need for Procuring Sleep.

Everything seemed to be of the best in the best of all possible worlds. Until my cough grew troublesome, my temperature rose slightly, and I could not sleep. Then I learned that while nothing matters very much to the patient who is comparatively well, and who is only kept in bed by a painless wound, many things, apparently trivial in themselves, matter a great deal to the patient who is irritable and rendered unduly sensitive by pain and discomfort. The essential thing in such cases is to make the patient comfortable and to procure sleep by the administration of a narcotic or anodyne. For some reason I was not at first given a morphia injection, my treatment consisting of hot compresses to the chest and medicine administered by the mouth. While the

* The writer is a young surgeon who was operated upon for appendicitis during the quiescent period. The operation was a success, and the after-course was uneventful except for a slight complication in the shape of a mild attack of broncho-pneumonia.

compresses eased me they did not make me sleep, and sleep was what I craved for. It came almost at once with an injection, and my experience has taught me that even in comparatively trifling cases it is better to procure sleep by such injection than to trifle and delay by trying other methods through that exaggerated fear of opium which still lingers in some medical minds.

Minor impressions that I garnered during those days when I was perhaps hypersensitive have reference to noise and nursing.

### The Distressing Effect of Noises.

The ordinary sounds in the room and outside it were magnified; the whistle of a passing train was almost a torment at times, and I can sympathise whole-heartedly with patients in a hospital whose wards overlook an unpaved street where motors and trams run all day long. The first essential of a ward is that it should be quiet and free from noise. Where this cannot be secured by a suitable site it must be sought for in another way, either by means of double windows or by making the walls as sound-proof as possible by aluminium sheeting or extra thickness. Noises within the ward should be reduced to a minimum. Again, I can appreciate the views of patients who dislike to have children in the wards; a crying child is an infliction which should be spared any hospital patient. The household noises, sometimes looked upon as inevitable, may, I feel sure, be avoided with proper care. The creaking lift or trolley, the ward wash-basin that makes its presence audible by the splash of the water, the door that moves dully on unoiled hinges, the coal-scuttle that rattles, the beds that make a monotonous chorus of semi-metallic expostulation when their occupants move, the window-blind that flaps, the nurse with leather-soled boots instead of felt slippers, the occasional fall of a piece of china or ward ware, and the loud conversation in the sisters' room—these are abominations that should not be tolerated in any circumstances. I had already learned to look askance on Christmas festivities in the ward, since my experience has taught me that where they give pleasure to three-quarters of the patients they are a source of irritation and mental and bodily discomfort to the other quarter, but I had scarcely realised before so acutely how trivial noises could make a patient miserable.

### Criticism of Ward Furniture.

With regard to nursing, my attention was speedily and unpleasantly directed to the absurdity of the ordinary model of urinal supplied to hospitals. This very necessary article of ward furniture is usually made far too small, while its shape is something that has been evolved by a nominalist who cannot have had much practical experience of its use. Hospital workers might usefully turn their attention to this utensil and try to evolve a model that is really serviceable—capacious, without being heavy, and of a shape that admits of its being handled by a patient who can in a measure help himself without the assistance of a nurse. This matter of the bed urinal is one of those trifling points which affords the patient extreme inconvenience and discomfort, but which he thinks is scarcely worth while complaining about, yet I feel sure that it is a point which ought to be considered in order to remedy the defect which at present undoubtedly exists.

Washing in the early morning was usually a comforting ritual, but I can understand patients who object to the process, especially when it is performed at a very early hour, with cold "wet" water that gives that unpleasant sticky feeling to which youngsters so vehemently object. Bed-making offers similar drawbacks, but here, as in other things, much depends on skilful nursing, and, as I have already said, I was fortunate in having nurses who were, in these things at least, the acme of perfection. A crease in the bed sheet may cause a patient far more discomfort than the wound or disease from which he suffers, and the attempt to put matters right, if made by a rough-handed nurse, may double the affliction. Proper warming and ventilation of the bedclothes are essential to the comfort of the patient, and in my case I had no reason to complain with regard to either. Nor had I any cause of complaint with regard to the food served. It was always brought in quantities just sufficient to tempt the appetite for more, always daintily and neatly served, and always cooked in a manner that reminded one of the best efforts of a good *chef*. These are important points from the patient's point of view, and they are often overlooked. Nothing, for example, can be more unattractive than the slabs of meat and masses of half-cold vegetables or pudding that I have seen my own patients consume in the wards when I was house surgeon. Then, I confess, I paid little attention to these matters, and perhaps would even have regarded a patient as cantankerous who complained. Now I know that complaint in such cases would have been perfectly reasonable and perfectly justifiable.

### A Last Impression.

My last impression concerns the time when I felt that I was fit and sufficiently well to be discharged. The room, notwithstanding its comfort and cheerfulness, had become irksome to me. I wanted to be away from hospital to recuperate elsewhere. This, I feel sure, is a want that many patients experience. The modern arrangement of building wards, especially on the surgical side, without day rooms, is to be commended. When a patient has no longer to lie abed he ought no longer to be in hospital; he should be sent to a convalescent home. The sojourn in hospital for three of four days after he is fit to go to such a home is quite needless and merely vitiates the hospital statistics by showing an unduly prolonged duration of treatment. In surgical cases this is especially to be deprecated. There are, of course, certain cases that need observation, but such observation may just as well be done at a convalescent home where there is a properly qualified medical man in charge.

